# Differential effects of received social support and mental health symptomology on affect in adults: an Ecological Momentary Assessment study

**DOI:** 10.1186/s12888-025-07117-3

**Published:** 2025-07-18

**Authors:** Tayler E. Truhan, Elyan Aarts, Emily McGlinchey, Talya Greene, Martin Robinson, Cherie Armour

**Affiliations:** 1 School of Psychology, Queen’s University Belfast, Belfast, UK; 2 Department of Clinical, Educational, and Health Psychology, University College London, London, UK

**Keywords:** Affect, Received social support, Mental health, Ecological momentary assessment

## Abstract

**Background:**

Perceived social support is robustly associated with affect and mental health. However, there is a relative lack of consensus regarding the effects of objectively received social support. Extant research is largely cross-sectional and thus potentially limited by recall bias around these time-varied variables.

**Methods:**

Addressing this, the current study employed Ecological Momentary Assessment (EMA) to examine the relationship between received social support, positive/negative affect, and mental health symptomology (PTSD, depressive, and anxiety symptoms). Trauma-exposed participants (*N* = 88) completed baseline assessments of mental health, and EMA of positive and negative affect and received instrumental and emotional support at 5 timepoints per day over a 7-day period. Analyses employed mixed effects modelling to assess the effect of received social support on affect over time for adults, and whether this association differed based on mental health symptomology. Clinical trial number: not applicable.

**Results:**

Results indicated received instrumental and emotional support were positively associated with positive affect. Average received instrumental support was positively associated with average negative affect, whereas recent received instrumental support was negatively associated with current negative affect. Findings did not significantly differ based on levels of mental health symptomology.

**Conclusions:**

Received emotional and instrumental support may therefore be related to both current and average mood, and be viable targets for intervention particularly for those with previous trauma exposure. Implications for improved understanding of the relationship between received social support and affect are discussed.

**Supplementary Information:**

The online version contains supplementary material available at 10.1186/s12888-025-07117-3.

## Introduction

Social support is instrumental in many aspects of one’s life, evidencing positive impacts on both physical and psychological health. Perceived social isolation has been linked to an increased risk for early mortality [1], stroke [2], and increased blood pressure [3]. On the psychological side, a lack of social support has been linked to higher risks for depression, posttraumatic stress disorder (PTSD), and anxiety [4], particularly for those that have experienced a traumatic event [5, 6]. Indeed, a lack of perceived social support has been robustly demonstrated to be among the most significant predictors of mental ill-health following trauma-exposure [7, 8]. Generally, social support is considered adaptive as it promotes sense of self and connection to others, improves the ability to cope with stress or traumatic events, and increases positive mood [9]. However, there are also instances in which social support can be maladaptive, if the support provided is considered intrusive or not needed [10, 11].

A distinction can be made between *received* and *perceived* social support. Received social support measures the specific supportive behaviors that are provided to individuals by their network. Perceived social support, on the other hand, focuses on the individual’s general perception on the availability and satisfaction of the support they receive [12]. There has been a relative lack of studies which have focused on received, rather than perceived, social support in relation to emotional well-being and mental health. Further, recall bias limits existing retrospective studies, as emotion is dynamic, such that it is continuously fluctuating and evolving over time [13]. The current study addresses this gap by utilizing ecological momentary assessment (EMA) methods to actively record and examine associations between received social support and positive or negative mood within a few hours and over time for trauma-exposed adults with and without symptoms of PTSD, depression, and anxiety.

Contrary to perceived support, evidence for the association between received social support, including instrumental (e.g., help with a task) and emotional (e.g., express care or concern) support, and emotional well-being has been mixed. One study found no association between received emotional or instrumental support and mood [14], whereas another found a negative association between received emotional support and negative mood, but no association between received instrumental support and either positive or negative mood [15]. Scholz and colleagues [16] suggested this may be due to age effects, as they found positive and negative associations between received emotional support and negative and positive affect, respectively, only for young adults, not older adults. Therefore, received instrumental and emotional social support may have differential effects on positive and negative mood [14, 15]. More recent EMA research on social interactions and mood indicates higher than usual social interactions are associated with increased positive mood, but unrelated to negative mood [17, 18]. In particular, more time than usual spent with friends, but not parents, is associated with increased positive affect [18].

Relational Regulation Theory posits affect (i.e., any experience of feeling or emotion; [19]) and affect regulation (i.e., the ability to alter one’s current emotions or thoughts and their expression; [20]) influence the relationship between social support and mental ill-health [21]. People with psychopathological disorders may experience heightened negative affect and lower positive affect. In depression, low positive affect manifests through central symptoms such as anhedonia (i.e., loss of pleasure; [22]). Individuals with PTSD may experience higher daily negative affect, particularly anger, which is compounded by a lack of confidence in their ability to regulate these emotions [23]. It is therefore important to understand the more fine-grained processes in the associations among received social support, affect, and mental health symptomology.

A limited number of studies have investigated the connection between mental health, affect regulation and social support, and most were based on singular or macro timescale (e.g., years) longitudinal self-report measures of perceived support [4]. A known limitation of retrospective self-report measures is that responses are often biased by feelings or memory [24]. A possible solution to this problem is the use of EMA. While EMA may still utilize self-report, this data is collected intensively, multiple times a day for an extended period, capturing responses either in-the-moment or related to the previous few hours. This all happens in the participant’s natural environment, resulting in high ecological validity and more accurate temporal measurement of time-variant affective states [25]. EMA produces a large set of observations related to subjective daily life experiences, which provides the possibility to detect small within-person changes in behavior and mood throughout the day [26]. Further, Relational Regulation Theory indicates the link between social support and mental health operates through affect regulation which occurs during “ordinary yet affectively consequential conversations about people’s daily lives, as well as in shared activities” [21], p.492), suggesting that EMA may be particularly suited to the study of received social support and affect states.

EMA has grown more popular in recent years, especially in the study of mental health. One EMA study focused on the connection between received social support (in-person time with friends relative to online video contact), depressive symptoms and affect during the COVID-19 pandemic [27]. They found participants with lower dysphoria symptoms (i.e., emotional and cognitive aspects of depression) experienced reduced negative affect after spending time with friends. This was not the case for participants with moderate to high dysphoria. However, the unique social situations created by the COVID-19 pandemic do not necessarily generalize to “normal” life, nor did this study include participants with anxiety or PTSD symptoms.

To address the mixed evidence regarding received support and emotional well-being, the current study examines the association between self-reported received instrumental and emotional social support and positive and negative mood in trauma-exposed adults using EMA methodology. We also include measurements of PTSD, anxiety, and depressive symptoms, to assess whether associations between received social support and mood change based on self-reported mental health symptomology. As previous research indicates perceived social support is protective for mental ill-health outcomes, we hypothesize that both recent (within-person) and average (between-person) self-reported received instrumental and emotional social support will be positively associated with in-the-moment and average positive affect and negatively with in-the-moment and average negative affect, although there is mixed evidence regarding received support. We also hypothesize that self-reported level of mental health symptomology will moderate the association between received social support and mood, such that there will be either a non-significant or possibly a negative association between received support and mood for individuals who reported above average levels of mental health symptomology.

## Methods

### Design

This study made use of an EMA design. Experiences over a 7-day period at 5-time points a day were sampled, meaning there was a possible total of 35 observations per participant. Notifications to respond to a brief survey were sent at pre-defined time points (9 am, 12 pm, 3 pm, 6 pm, 9 pm). This was done to promote higher compliance, as fixed time assessments are predictable and less likely to be missed [28]. Clinical trial number: not applicable.

### Sample

There were initially 945 participants who provided consent. A large proportion of responses were removed due to fraud scores greater than or equal to 30 (computed with Qualtrics inbuilt fraud detection; *N* = 236), participants who did not provide their e-mail to enroll in the EMA study (*N* = 335), duplicate entries (*N* = 79), and participants who never started the EMA study (*N* = 205). The final sample comprised 90 participants from the UK, USA and the Netherlands with a history of trauma participated in the study over 7 days, from March 2022 – January 2023. Most participants identified as female (75.6%), between the ages of 18 and 24 (50%). A complete overview of sociodemographic variables can be found in Table 1. To be included in the study, participants needed to have experienced at least one traumatic event in their lifetime, be at least 18 years old, own a smartphone with access to the internet, and be proficient in the English language.

**Table 1:** Demographic information

Sociodemographic variables	N	% of occurrence in sample
Gender		
Cisgender Female	68	75.6%
Cisgender Male	19	21.1%
Non-conforming	2	2.2%
Transgender Man	1	1.1%
Age, years		
18–24	45	50%
25–34	26	28.9%
35–44	11	12.2%
45–54	5	5.6%
55–64	3	3.3%
Place of residence		
United Kingdom	71	78.9%
United States	9	10%
Netherlands	9	10%
Other	1	1.1%
Employment		
Student	41	45.6%
Employed	39	43.3%
Unemployed	6	6.7%
Retired	2	2.2%
Furloughed	1	1.1%
Other	1	1.1%
Ethnicity		
White	71	78.9%
Black/African/Caribbean/Black British	5	5.6%
Asian/Multiple Ethnic groups	5	5.6%
Asian/British	4	4.4%
Middle Eastern/North African	4	4.4%
Other	1	1.1%
Relationship status		
Single or never married	56	62.2%
Married or living with partner	22	24.4%
Separated or divorced	2	2.2%
Widowed	3	3.3%
Prefer not to say	1	1.1%
Other	6	6.7%
Highest form of completed education		
Left school/no qualifications	8	8.9%
Secondary school or equivalent	28	31.1%
Associate’s degree or equivalent	5	5.6%
Bachelor’s degree	22	24.4%
Master’s degree	22	24.4%
Doctoral degree/PhD	4	4.4%
Missing	1	1.1%

Total *N* = 90. All nations considered part of the United Kingdom have been collapsed into the category UK. These include Northern Ireland, England, Scotland, Wales, Other (in the UK)

### Missing data

There were 90 adults that agreed to participate in the study and all were included. Participants were included even if they did not respond to 100% of beeps, as mixed effects models are robust against missing data [28]. Participants responded to an average of 85% of beeps (range 54–100%). There was a total of 2,687 observations included in the current study.

### Procedures

This study was conducted in accordance with the Declaration of Helsinki and was approved by the host university’s Faculty of Engineering and Physical Sciences Research Ethics Committee (ref: *redacted for masked review*). The study was advertised via social media posts, and via flyers distributed throughout the host university. The flyer referred to the ‘ability to cope with stressful events’ rather than trauma in order not to deter potential participants who might feel that “trauma” is too extreme to characterize their experiences. The link in the advertisement directed participants to an online screener survey. They were presented with the information letter, informed consent, and the Life Events Checklist for DSM-5 [29]. All participants provided consent. Those reporting past trauma (either one event or multiple events) were screened into the study. Eligible participants were immediately referred to the baseline assessment on their own devices. The survey included measures of the PTSD checklist for DSM-5 criteria (PCL-5; [30]), the Patient Health Questionnaire (PHQ-9; [31]), and the brief self-report scale for Generalized Anxiety Disorder (GAD-7; [32]). Participants were sent extensive information videos on how to download the Avicenna (https://avicennaresearch.com/) app onto their personal mobile device, make an account and complete the EMA study.

The day following completion of the baseline assessment began the 7-day EMA assessment. Participants received five prompts to complete a survey (5–7 min) every 3 h from 9 am until 9 pm on their smartphone. Participants received a reminder/feedback on their compliance when they were close to dropping below the cut-off for compensation (which was 50%). After completion of the 7-day period, participants received an incentive based on the number of surveys they completed (£5 Amazon voucher for 50% completion, £10 for 80%, and £15 for 100%).

### Measures

#### Traumatic events

In the baseline questionnaire, participants were asked to indicate how many events they had experienced from the Life Events Checklist for DSM-5 (LEC-5; [29]). The LEC-5 contains a list of 17 potentially traumatic events (e.g., life-threatening illness, physical assault, sexual assault, natural disasters), with a binary (0 = *no*, 1 = *yes*) endorsement of experience of these events at any time in one’s life. The LEC-5 demonstrates excellent reliability and validity [29].

#### Mental health symptomology

PTSD symptoms were measured using the PCL-5 [30]. PTSD diagnostic grouping is best predicted by a cut-off score of 31 or above (*max* = 80) with a specificity of.95 and sensitivity of.83 [33]. Depression symptoms were assessed with the PHQ-9 [31]. A cut-off score of 10 or above (*max* = 27) indicates moderate depression symptoms with a sensitivity and specificity of.88 [31]. General anxiety symptoms were checked with the GAD-7 [32]. At a cut-off score of 8 or above (*max* = 21), the GAD-7 has a sensitivity of.77 and a specificity of.82 for identifying any anxiety disorder [32]. Reliability estimates, as McDonald’s omega, were high in the current study for PTSD (ω = 0.95), depression (ω = 0.92), and anxiety (ω = 0.90) symptoms. Mental health symptomology was included as a singular continuous variable comprising the sum of PCL-5, PHQ-9, and GAD-7 scores.

#### Positive and negative affect

EMA survey items included five positive (Happiness, calm, enthusiasm, hope, and optimism) and five negative affect ratings (Anger, fear, guilt, shame, and sadness) based on previous EMA studies [34, 35]. Participants rated all ten affect items during each ‘beep’ of the EMA study. These items were rated on a Likert scale from 1 (*Not at all*) to 5 (*Extremely*). Participants were asked to rate their affect ‘right now’. Items were used to create a mean score for positive and negative affect in-the-moment and over the course of the study (*Range* = 1–5). Between- and within-person reliability estimates were high for between-person positive (ω = 0.96) and negative affect (ω = 0.97), and within-person positive (ω = 0.80) and negative affect (ω = 0.73).

#### Received instrumental and emotional social support

Recently received instrumental and emotional social support were measured with one item each based on an EMA study by Vella and colleagues [36], namely, “In the last three hours, someone has helped me with an errand/task” (instrumental) and “In the last three hours, someone has expressed care/concern for me” (emotional). These items were rated on a Likert scale from 1 (*Not at all*) to 5 (*Extremely*).

#### Time

Participants could complete the affect and received social support questionnaires a total of 35 times over 7 days (5 time-based surveys per day). Each survey was coded cumulatively, so that the first survey they received was one, and each survey after that increased by one whether they completed the survey or not. This way we can account for time for each participant over the course of the study. The assessment number was used as a proxy for time in the negative and positive affect models.

### Statistical analyses

Within- and between-group correlations were calculated using the *multilevel.cor* function in R Studio version 2024.12.0. As EMA data inherently has a hierarchical structure in which repeated assessments (Level 1) are nested within individuals (Level 2), mixed effects models can be used to extricate individual-level variability in positive and negative affect. Mixed effects models are also particularly suited to analyzing EMA data as they include additional random effects which account for individual-level differences [28]. In the current study, mixed effects models were estimated with the *nlme* package version 3.1–157 [37]. The estimation method for all models was set as restricted maximum likelihood. First, we calculated the Intra-Class Correlation (ICC) for positive and negative affect and received instrumental and emotional social support to identify the within- and between-person variance. ICCs higher than 0.5 indicate that between-person differences are higher than within-person differences. In EMA, variables should not exhibit overly high ICCs or they are not suited to the study of within-person variability (e.g., we would not expect intelligence to vary day by day). Second, we examined separate within- and between-subjects models for positive and negative affect, with average received instrumental and emotional social support and within-person mean-centered received instrumental and emotional social support as predictors.

Finally, we conducted a series of mixed effects models for both positive and negative affect. Akaike Information Criterion (AIC) and Bayesian Information Criterion (BIC) values were utilized to identify the model with the superior fit, with lower values indicating a better fit. Raftery [38] recommends that BIC differences larger than 10 are indicative of a superior model. We also tested whether subsequent models fit better using Likelihood Ratio Tests (LRT). Received social support was within-person mean centered for all models. Model 1 included only *received instrumental or emotional social support*. Model 2 included *received instrumental or emotional social support,* and *time* (assessment number). We also tested the influence of time measured as beep/alert number (i.e., 1–5 in the current study) and participant response time in hours since midnight, but neither had a significant effect in the models. Model 3 included *received instrumental or emotional social support*, *time*, and *mental health symptomology*. Model 4 included *received instrumental or emotional social support*, *time*, *mental health symptomology*, the *Level 1 interaction* between received instrumental or emotional social support and time, and the *cross-level interaction* between received instrumental or emotional social support and mental health symptomology. In Model 5, we included *autocorrelation of Level 1 errors*, which accounts for temporal dependencies in EMA data, as we assume Level 1 errors follow a first-order autoregressive process [39, 40]. See Supplementary Materials Fig. 1 A-E for a depiction of the series of models tested. A simulation study of statistical power in two-level models suggests a ‘rule of thumb’ for sample size to be at least 125 participants with 25 measurement occasions to achieve 80% power to detect medium cross-level interactions between two continuous predictors (with level 1 and 2 direct effects requiring lower sample sizes; [41]). Although we had slightly less overall participants, our study contained 10 additional measurement occasions (total = 35).

### Transparency and openness

We report sample size and characteristics, information on the power of our sample size to detect the different effects tested, handling of missing data (Sect.“ Missing data”), all manipulations, and all measures in the study. Analysis code and output is available at (https://osf.io/scjpm/). This study’s design and its analysis were not pre-registered. Data is available upon reasonable request from the corresponding author.

## Results

### Descriptive statistics

On average, females had 0.45 (*p* =.035) lower positive affect and 0.34 (*p* <.001) higher negative affect. There was no significant effect observed of age on positive or negative affect. Within- and between-person correlations are presented in Table 2. Experience of sexual assault or another uncomfortable or unwanted sexual encounter was most frequently rated as the index event, i.e. that bothered participants most (32% of participants), out of all traumatic events they had experienced. Participants indicated that they had experienced an average of 4.37 potentially traumatic events (*SD* = 3.26, *Max* = 16), 58% met criteria for a mental health condition (37% depression, 44% anxiety, and 51% PTSD), and 42% experienced co-morbid mental health conditions. Participants who met criteria for a mental health condition reported an average PTSD score of 42.58 (*SD* = 13.40), average anxiety was 10.69 (*SD* = 4.19), and average depression was 12.38 (*SD* = 6.17). Participants who did not meet criteria for a mental health condition reported an average PTSD score of 11.11 (*SD* = 7.19), average anxiety was 2.79 (*SD* = 2.06), average depression was 3.18 (*SD* = 2.33). Total self-reported mental health symptomology ranged from 2 to 108.

**Table 2:** Correlations for within-group and between-group variables in the current study

***Within-Group***							
		Instrumental Support		Emotional Support		Positive Affect	
Emotional Support		0.38^***^					
Positive Affect		0.19^***^		0.16^***^			
Negative Affect		−0.07^**^		0.00		−0.44^***^	
***Between-Group***							
	Inst. support	Emo. support	Pos. affect	Neg. affect	PTSD symp	Anx. symp	Dep. symp
Emo. support	0.78^***^						
Pos. affect	0.38^**^	0.40^***^					
Neg. affect	0.26	0.08	−0.41^***^				
PTSD symp	0.18	0.04	−0.37^***^	0.57^***^			
Anx. symp	0.05	−0.06	−0.47^***^	0.54^***^	0.76^***^		
Dep. symp	0.03	−0.08	−0.52^***^	0.58^***^	0.83^***^	0.82^***^	
MH total	0.13	0.00	−0.44^***^	0.60^***^	xx	xx	xx

*Inst* instrumental, *Emo* emotional, *Pos* positive, *Neg* negative, *Symp* symptoms, *Anx* anxiety, *Dep* depressive, *MH* mental health

^***^p <.001, ^**^p <.01

Average positive and negative affect were 2.95 (*SD* = 0.73) and 1.46 (*SD* = 0.55), respectively. Average received instrumental and emotional support were 2.01 (*SD* = 0.72) and 2.18 (*SD* = 0.84), respectively. There were 1,250 and 1,039 instances in which participants reported not receiving any instrumental or emotional support, respectively, in the last three hours. For both positive and negative affect, approximately 40% of the variance was due to within-person differences (ICC), whereas 60% was due to between-person differences. For received emotional social support, 53% of the variance was due to within-person differences. For received instrumental social support, 64% of the variance was due to within-person differences. Therefore, within-person and between-person differences were approximately similar in the current study, except for instrumental social support which exhibited the highest within-person variability.

### Within- vs. between-subjects received social support and affect

Individuals who reported higher than average received emotional social support also reported higher average positive affect (*p* <.001). Further, when individuals reported higher than average recently received emotional social support, they also reported higher in-the-moment positive affect compared to their average levels that week (*p* <.001). Individuals who reported higher than average received instrumental social support also reported higher average positive affect (*p* <.001). Further, when individuals reported higher than average recently received instrumental social support, they also reported higher positive affect than average in these moments (*p* <.001).

Neither average nor recent received emotional support were significantly associated with average or momentary negative affect. However, individuals who reported higher than average received instrumental social support also reported higher average negative affect (*p* =.026). However, when individuals reported higher than average recently received instrumental social support, they reported lower negative affect in these moments (*p* =.014).

### Mental health symptomology, received social support, and positive affect

Model fit values (AIC, BIC) of all models are presented in Table 3. For the models with received *emotional* social support, AIC and BIC and a LRT indicated Model 4 (inclusion of support-time and support-mental health interactions) was not a superior fit to Model 3. In Model 4, there was no significant difference in the slopes of individuals reporting high versus low mental health symptomology, with both groups showing a positive association between emotional support and positive affect. The Level 1 interaction between time and received emotional support was also not significant. Therefore, Model 3 was refit to account for the effects of predictors at the previous time point. Model 5 (see Table 4) had the best fit according to AIC and BIC values and a LRT. Time was negatively associated with positive affect, such that positive affect decreased the longer the individual participated in the study, which may be indicative of initial elevation bias [42]. Individuals who reported more mental health symptomology reported significantly lower positive affect. The inclusion of autocorrelated residuals indicated the effect of the predictors on positive affect spilled over into the next assessment window.

**Table 3:** Fit indices of mixed effects models for received social support and affect

***Received emotional social support and positive affect***					
	**Model 1**	**Model 2**	**Model 3**	**Model 4**	**Model 5**
**Predictors**	Emotional Support	Emotional Support, Time	Emotional Support, Time, MH symptom	Emotional support, Time, MH symptom, interactions	Emotional support, Time, MH symptom, Autocorrelation
**AIC**	4824	4773	4765	4793	4661
**BIC**	4859	4832	4830	4869	4732
***Received instrumental social support and positive affect***					
	**Model 1**	**Model 2**	**Model 3**	**Model 4**	**Model 5**
**Predictors**	Instrumental Support	Instrumental Support, Time	Instrumental Support, Time, MH symptom	Instrumental support, Time, MH symptom, interactions	Instrumental support, Time, MH symptom, Autocorrelation
**AIC**	4812	4758	4751	4777	4653
**BIC**	4847	4817	4816	4854	4724
***Received instrumental social support and negative affect***					
	**Model 1**	**Model 2**	**Model 3**	**Model 4**	**Model 5**
**Predictors**	Instrumental Support	Instrumental Support, Time	Instrumental Support, MH symptom	Instrumental Support, MH symptom, interactions	Instrumental Support, MH symptom, Autocorrelation
**AIC**	3186	3167	3161	3176	3050
**BIC**	3221	3226	3202	3223	3097

*MH* mental health

**Table 4:** Random intercepts and slopes autoregressive model for received social support and positive affect

	**Coefficients**	**SE**	**95% CI**	**t-value**
***Received Emotional Support***				
**Fixed Effects**				
Intercept	3.52	0.13	3.27, 3.77	27.49^***^
Emotional Support	0.10	0.02	0.07, 0.13	6.72^***^
Time	−0.004	0.002	−0.007, −0.001	−2.57^*^
MH Symptom	−0.01	0.002	−0.02, −0.006	−4.69^***^
**Random Effects**		**Correlations**		
Intercept SD	0.63	Inter	Time	
Slope (emo. support) SD	0.08	0.07	−0.51	
Slope (time) SD	0.01	−0.02		
Residual	0.55			
Autocorrelation	0.22			
***Received Instrumental Support***				
**Fixed Effects**				
Intercept	3.51	0.13	3.26, 3.76	27.26^***^
Instrumental Support	0.11	0.01	0.08, 0.13	8.35^**^
Time	−0.004	0.002	−0.007, −0.0008	−2.43^*^
MH Symptom	−0.01	0.002	−0.02, −0.006	−4.58^***^
**Random Effects**		**Correlations**		
Intercept SD	0.64	Inter	Time	
Slope (inst. support) SD	0.06	0.005	−0.60	
Slope (time) SD	0.01	−0.08		
Residual	0.54			
Autocorrelation	0.21			

*Emo* emotional, *Inst* Instrumental, *Inter* Intercept, *MH* mental health, *SD* standard deviation

^*^p <.05, ^**^p <.01, ^***^p <.001

For the models with received *instrumental* social support, in Model 4, there was no significant difference in the slopes of individuals who reported high versus low mental health symptomology, with both groups showing a positive association between received instrumental support and positive affect. There was also not a significant interaction between time and received instrumental support. AIC and BIC and a LRT indicated Model 4 was not a superior fit to Model 3. Therefore, Model 3 was refit to account for the effects of predictors at the previous time point. Model 5 (see Table 4) had the best fit according to AIC and BIC values and a LRT. The inclusion of autocorrelated residuals indicated that the influence of predictors carried forward into the next assessment time.

### Mental health symptomology, received social support, and negative affect

For the models with received *emotional* social support, of the effects tested, only mental health symptomology was significantly associated with negative affect.

For the models with received *instrumental* social support, time was not significantly associated with negative affect in Model 2. Therefore, this model was rejected, and Model 1 was refit to include mental health symptomology (Model 3). In Model 4, there was no significant difference in the slopes of individuals who reported high versus low mental health symptomology, with both groups showing a negative association between recent instrumental support and momentary negative affect. AIC and BIC and a LRT indicated Model 4 was not a superior fit to Model 3. Therefore, Model 3 was refit to account for the effects of predictors at the previous time point. Model 5 (see Table 5) had the best fit according to AIC and BIC values and a LRT. Mental health symptomology was significant, such that individuals who reported higher mental health symptomology reported significantly higher negative affect. The inclusion of autocorrelated residuals indicated that the influence of predictors carried forward into the next assessment time.

**Table 5:** Random intercepts and slopes autoregressive model for received instrumental support and negative affect

	**Coefficients**	**SE**	**95% CI**	**t-value**
**Fixed Effects**				
Intercept	0.96	0.09	1.06, 1.39	11.25^***^
Instrumental Support	−0.03	0.01	−0.05, −0.007	−2.64^**^
MH Symptom	0.01	0.002	0.20, 0.64	6.95^***^
**Random Effects**		**Correlations**		
Intercept SD	0.44	Inter		
Slope (inst. support) SD	0.05	−0.34		
Residual	0.41			
Autocorrelation	0.22			

*Inst* Instrumental, *Inter* Intercept, *MH* mental health, *SD* standard deviation

^**^p <.01, ^***^p <.001

## Discussion

This study investigated the influence of average and recent self-reported received instrumental and emotional social support on average and in-the-moment positive and negative affect, and whether these associations changed for people based on self-reported levels of PTSD, anxiety, or depressive symptomology. Differential associations were observed between received social support and affect, depending on time (recent versus average support), type of received support (instrumental versus emotional), and affect (positive versus negative). Findings of the current study corroborate Relational Regulation Theory [21] regarding *received* social support, such that the link between received social support and improved mood was particularly evident ‘in the moment’, rather than over time, suggesting ordinary, daily supportive social interactions may be particularly beneficial for mental health and well-being. We were able to make comparisons between recent and average received support and momentary affect using EMA methodology, which allowed us to empirically test the predictions of Relational Regulation Theory.

Higher *perceived* social support has been consistently associated with improved well-being and mental health [4, 5]. Our results suggest positive social interactions (i.e., received social support) are positively associated with positive mood, including feelings of happiness, hope, and optimism, thereby supporting our hypotheses regarding the positive association between received instrumental and emotional social support and positive affect. Further, increased emotional and instrumental support that is occurring both within a few hours and over time may co-occur with positive mood. It is worth noting that received emotional support was somewhat subjective in the current study, as participants were asked whether ‘someone expressed care/concern’. Therefore, it is possible that participants who experience higher positive mood interpret or perceive the recent actions of others as more emotionally supportive.

Previous daily diary research found received emotional social support was negatively associated with positive affect for young people (younger than 40 years) whereas it was positively associated with positive affect for older people (older than 61 years; [16]). Our results contradict this previous research, especially considering 92% of our sample was younger than 44 years. However, in another daily diary study with couples coping with multiple sclerosis, receiving emotional social support was positively associated with positive end-of-day mood [15]. As our sample comprised adults who have previously experienced a traumatic event, and Kleiboer and colleagues [15] included couples coping with a chronic illness, it may be receiving social support is particularly beneficial regarding positive mood for adults with adverse or traumatic experiences.

Other EMA parenting research examining parent-adolescent interaction quality and adolescent mood has found there can be heterogeneity in the relation between interaction quality and adolescent affect across families [10, 43]. It is likely that this same heterogeneity exists regarding received social support and affect – such that, the within-person effects of received social support on affect may differ from person to person. While for the majority of our sample there was a positive association between recently received emotional and instrumental support and momentary positive mood, a smaller portion of the sample may have experienced a negative association or even no association at all. Therefore, future research might also examine between-person differences in within-person associations of received social support on mood.

However, results regarding negative affect refuted our hypotheses in several ways. Self-reported received emotional social support was not associated with reduced negative affectivity, rather there was no significant association between recent or average received emotional social support and momentary negative affect, and average instrumental support was positively associated with momentary negative affect. This result may be attributed to a spurious relationship, whereby those who report more frequent negative affect seek or receive more instrumental support from others. Typical patterns of received support and engagement may mute effects in the current study leading to the finding that emotional and instrumental support were associated with null and counter-intuitive effects respectively. These results are contrary to evidence which suggests emotional support is positively [16] or negatively associated with negative affect (in patients but not in romantic partners, [15]), and instrumental support is unrelated to negative affect [15]. Another study with couples undergoing in-vitro fertilization found a non-significant association between received emotional and instrumental social support and negative affect, but a significant negative association between *providing* emotional support and negative affect for women [14]. Reciprocal or mutual emotional support may be more impactful on negative affect particularly.

Interestingly, we found that recent, rather than average, self-reported instrumental support was negatively associated with momentary negative affect, indicating there are differential associations between instrumental support and negative affect between and within individuals. These results highlight a need to examine both within- and between-person associations to understand the relation between instrumental support and negative affect, a key benefit of the EMA methodology used in the current study. Melrose and colleagues [11] suggested that a negative or lack of association between received support and well-being stems from whether the individual feels that the received support is needed or not. When the need for support was considered, stronger associations were observed between received support and mental health outcomes. Relational Regulation Theory also posits that the relative importance of the provider of support to the recipient (e.g., a close friend versus an acquaintance) plays an important role [21]. Therefore, it may be that receiving instrumental support when it is needed, particularly from someone emotionally close to the recipient, is helpful and associated with lower negative mood. However, higher-than-average instrumental support over time may be associated with feelings of inadequacy or anger. These predictions should be tested in future research.

Even though greater mental health symptomology was associated lower and higher on average positive and negative affect, respectively, associations between received social support and affect were not significantly different between people who reported higher versus lower mental health symptomology, contrary to our hypotheses. These results support other EMA research in this area indicating the presence of others, particularly friends, improves positive mood across all levels of depression [27, 44], but contradict findings showing the presence of friends reduces negative affect only for those reporting low dysphoria [27]. A systematic review and meta-analysis of EMA studies on social interactions and mood found the presence of others, particularly close social relations, is beneficial for positive mood in general population samples – but has variable effects on negative mood [45]. However, the aforementioned studies assessed the presence or absence of friends, not specific received support. Individuals with symptoms of depression, anxiety, or PTSD might experience the same benefit on positive mood (as individuals reporting low symptoms) of interactions characterized as receiving social support, rather than just being in the presence of a friend. It might therefore be of pertinence for future research to determine whether it is received social support or social interactions that leads to more positive mood.

Experimental evidence suggests in the moment social support is more well-received if it is ‘invisible’ – such that the support provided does not make the recipient feel inefficacious or incapable [46, 47]. Therefore, fostering a greater acceptance of and openness towards support, as well as promoting ways to access more positive social interactions, such as community mental health support groups, may be particularly beneficial for people who have experienced a traumatic event. Receiving social support even long after a traumatic event can be beneficial to mental health outcomes [5]. Further, in couples or family counseling, practitioners could provide educational materials on adaptive forms of support which do not make the recipient feel criticized and highlight the potential negative impacts of ‘over-helping’ the recipient when it is not truly needed. Negative social reactions from others can have long-lasting deleterious impacts on an individual’s adjustment to trauma and trauma disclosure [5].

There are several limitations to the current study that warrant consideration. Although we were able to assess associations between recently received social support and momentary mood, as well as associations over time, we cannot infer causation due to the nonexperimental nature of the study and because we did not examine temporal relations between these constructs. Further, we did not examine gender or age as moderators due to the small sample size of males (*N* = 20) and older adults (over age 44: *N* = 7) in the study sample. This means that it was not possible to disentangle the potentially separate associations between received support and mood in males and females [14], or younger and older adults [16]. Our findings regarding received social support and mood may not be generalizable to all genders, races, or ethnicities, as our sample was primarily young to middle-aged White females. The study employed a fixed time-point design for the EMA surveys. While this approach ensures higher compliance than other approaches (e.g., random time-points, semi-random time-points), it has somewhat less ecological validity as participants may expect the EMA assessments. Lastly, the item measuring received emotional support requires the participant to make a subjective interpretation, so it should be considered that our assessment of emotional support may not be entirely objective. Future research might utilize EMA with a larger more diverse sample to examine age and gender effects on both received and perceived social support within a few hours and over time, and how these associations operate across races and ethnicities.

## Conclusions

This study adds to the growing body of literature which attempts to understand the mixed findings regarding the beneficial or deleterious effects of received social support and provides empirical support for Relational Regulation Theory. Emotional and instrumental support exhibit differential associations with positive and negative mood. Results indicate received emotional and instrumental support may co-occur with positive mood, and therefore may be particularly beneficial to foster for those with previous trauma exposure, regardless of mental health symptomology, through community support groups or other programs. Further, an individual’s need for support, especially practical instrumental support, should be taken into consideration as repeatedly receiving instrumental support may co-occur with higher negative mood, whereas momentary instrumental support, perhaps when it is really needed, may co-occur with lower or no negative mood. Future work may use methods which offer increased ecological validity, such as EMA, to study the interplay of different types of social support and emotional well-being for people with mental health symptomology.

## Supplementary Information

Supplementary Material 1.

## Data Availability

Data is available upon reasonable request from the corresponding author.

## Abbreviations

AIC: Akaike Information Criterion
BIC: Bayesian Information Criterion
EMA: Ecological Momentary Assessment
ICC: Intra-Class Correlation
LRT: Likelihood Ratio Test
PTSD: Post-Traumatic Stress Disorder
