# Supplementary material for: Differential effects of received social support and mental health symptomology on affect in adults: an Ecological Momentary Assessment study
Source: BMC Psychiatry. 2025 Jul 18;25:713. doi: 10.1186/s12888-025-07117-3 (PMC12272982; doi:10.1186/s12888-025-07117-3)
Supplement: Supplementary file 1 — Supplementary Material 1. [file 12888_2025_7117_MOESM1_ESM.docx]

**Differential effects of received social support and mental health symptomology on affect in adults: An Ecological Momentary Assessment study**

**SUPPLEMENTARY MATERIALS**

**
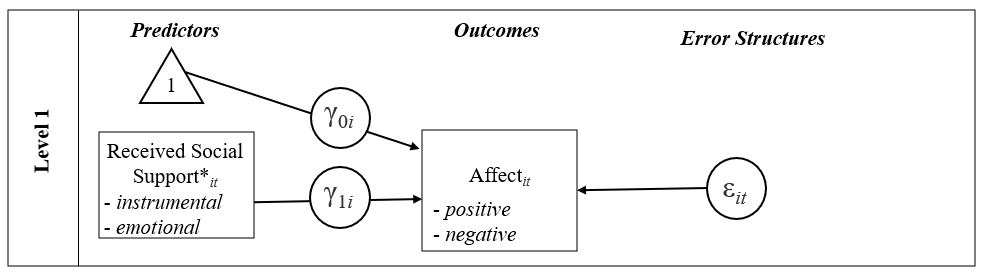
A.**

**
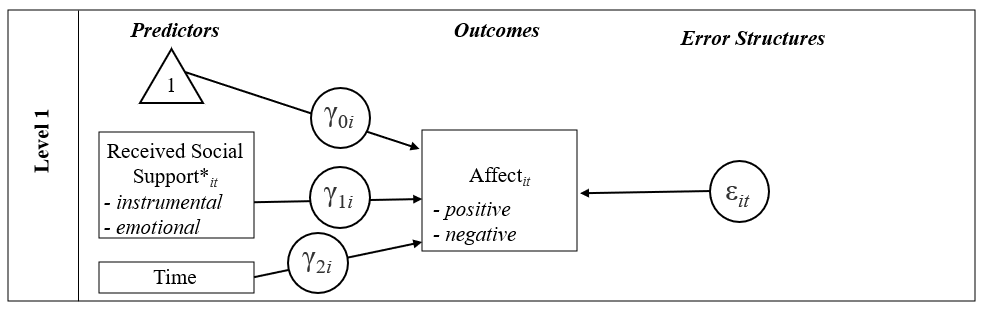
B.**

**
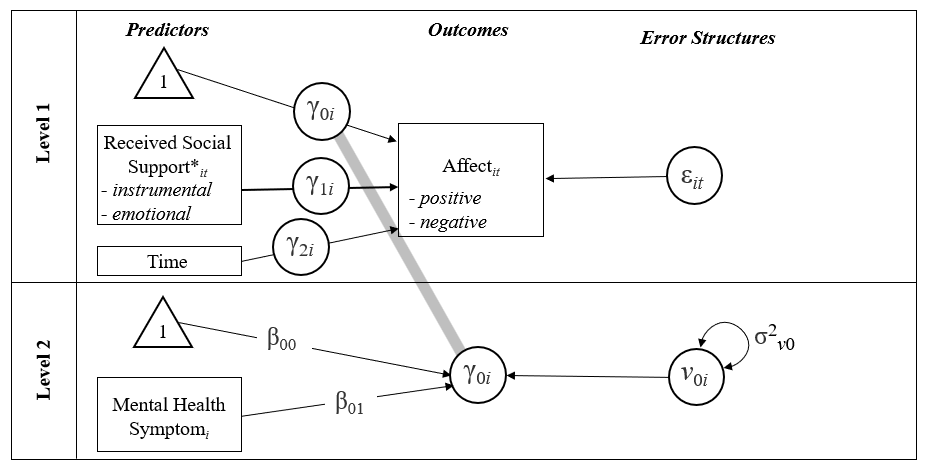
C.**

**
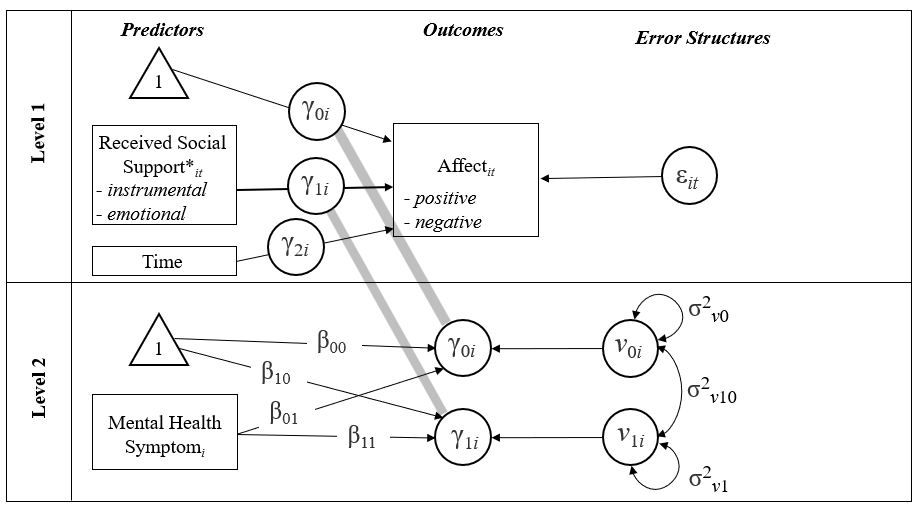
D.**

**
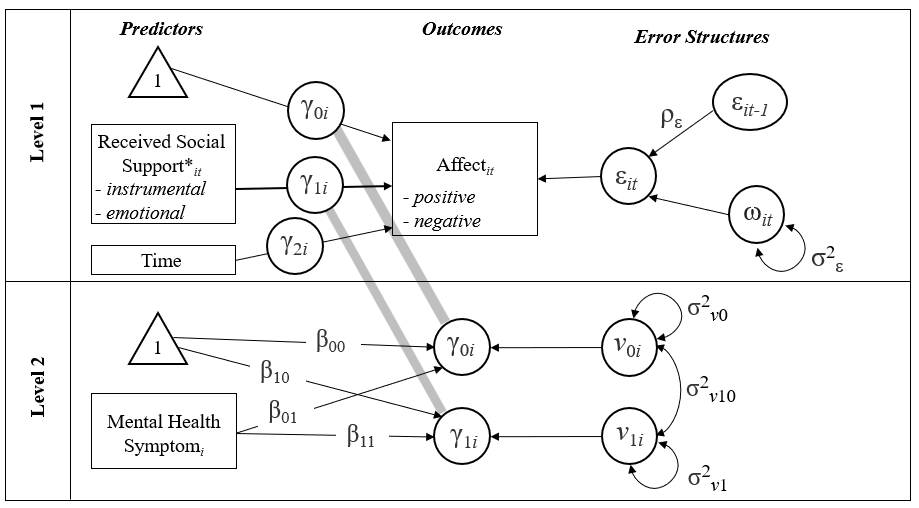
E.**

**Figure 1. A.** *Model 1: Received social support and affect.* **B.** *Model 2: Received social support, time, and affect.* **C.** *Model 3: Received social support, time, mental health symptomology, and affect.* **D.** *Model 4: Received social support, time, and the cross-level interaction between mental health symptomology and received social support. The Level 1 interaction for time and received social support is not included in the figure for clarity.* **E.** *Model 5: Assume the Level 1 errors* (*ε_it_*) *follow a first-order autoregressive process.*
